# Assessment of peri‐urban livestock producers’ willingness to pay for improved forages as cash crops

**DOI:** 10.1002/agj2.20953

**Published:** 2022-01-28

**Authors:** Adama Ouédraogo, Nouhoun Zampaligré, Balehegn Mulubrhan, Adegbola T. Adesogan

**Affiliations:** ^1^ Institut de l'Environnement et de Recherches Agricoles Station de Farako‐Ba Bobo Dioulasso Burkina Faso; ^2^ Feed the Future Innovation Lab for Livestock System Univ. of Florida Gainesville FL 32611 USA

## Abstract

Availability of quality feed is a major constraint for livestock production in Burkina Faso. Despite previous efforts to test improved forages at research stations to overcome the dry‐season feed gap, little has been done to promote them as cash crops that can contribute to meeting the growing feed demand in the country. This study was undertaken to evaluate the willingness to pay (WTP) for improved forage by livestock producers in the peri‐urban livestock production systems of Burkina Faso. A total of 202 livestock producers were interviewed using semi‐structured questionnaires. The contingent valuation method and Tobit econometric model were used to analyze the survey data. Exactly 79% of the interviewed livestock producers were willing to pay for improved forages for their livestock. Key factors that significantly affect this decision were the price of cottonseed cakes used as supplemental feed (*P *= .001), farmers’ knowledge about improved forage crops (*P *= .001), farmers’ ethnicity (*P *= .05), and farmers’ practice of daily grazing and transhumance (*P *= .01). The estimated WTP for improved forage as a cash crop was US$0.32 kg^–1^ for all livestock producers and $0.58 kg^–1^ for those who only expressed a positive WTP. The positive WTP for improved forages and factors affecting that decision suggest that producing improved forages is a viable alternative to expensive cottonseed cakes and the practice of transhumance to overcome the dry‐season feed gap. Therefore, dissemination of improved forages is recommended to market‐oriented crop farmers to meet the growing feed demand in Burkina Faso.

## Core Ideas

- Livestock keepers in urban areas of Bobo‐Dioulasso are willing to pay for cultivated fodder.
- Willingness to pay for improved fodder is affected by the price of cottonseed cake.
- Producing quality fodder as a cash crop is an alternative for bridging the recurrent feed gap.
- Dissemination of improved forages to market‐oriented crop farmers is recommended.

**Abbreviations**
ASF: animal‐sourced food
CVM: contingent valuation method
WTP: willingness to pay.

## 1 INTRODUCTION

According to the United Nations Population Fund (UNFPA, 2017), the world is experiencing rapid human population and urban growth. More than half of the world's population today lives in cities, and this number will reach about 5 billion by 2030. This urbanization will mainly affect Africa and Asia, where it will cause considerable disruption to food, socio‐economic, and environmental issues. According to Burkina Faso National Institute of Statistics and Demography (INSD, 2017), the country's population was estimated to be about 21 million inhabitants in 2020. This demographic growth implies high demand for food, including animal‐sourced food (ASF) (e.g., milk, meat, and eggs), which will be needed to meet the nutrient requirements of this population. Herrero et al. (2014) suggested that the demand for animal products will double or triple by 2050 for sub‐Saharan Africa. In Burkina Faso, demand for meat will be in the order of 940,556 t yr^–1^, and milk demand will amount to 68,286 t yr^–1^ by 2025 (Da, 2015).

The demand for ASF is an opportunity for the livestock sector in sub‐Saharan Africa, but some constraints need to be overcome to leverage this opportunity. One of the main constraints of livestock production in sub‐Saharan Africa in general and particularly in the Sahel and Burkina Faso is the low year‐round availability and access to quality feed resulting in low reproduction rates and low dairy and meat production (MRA, 2015). As reported by Gebremedhin et al. (2003), sub‐Saharan Africa faces a nutritional and feed imbalance in the livestock sector.

In Burkina Faso, the majority of livestock (cattle [*Bos taurus indicus*], goats [*Capra aegagrus hircus*], and sheep [*Ovis aries*]) rely on the fluctuating supply of naturally occurring pasture resources and the use of crop residues as year‐round feed. However, due to cropland expansion, recurrent droughts, and inappropriate cropping and grazing management, there is decline in pasture and rangeland productivity (Kiema et al., 2014). This, coupled with the increase of the countries’ livestock numbers (MRAH, 2019), results in year‐round recurrent feed gaps both in quantity and quality.

To mitigate livestock feed constraints, various strategies and practices are used by farmers and policy makers. Increased use of crop residues (cereal stover and legume haulms), dry season (January–May) supplementation with browse and agricultural byproducts, and increased transhumance, both in country and transboundary toward Ghana, Togo, Bénin Republic, and Republic of Côte d'Ivoire, are the main strategies and practices adopted by livestock producers to feed their animals year round (CORAF/WECARD, 2015; Kiema et al., 2014). The latter traditional animal feeding practice is a source of violent conflicts over access to pasture resources between crop farmers and pastoralist communities along the transhumance route (CORAF/WECARD, 2015), despite the existence of national and regional regulations to facilitate herd movement and access to water and fodder (Kiema et al., 2012).

Imports and production of commercial feeds and agricultural byproducts for dairy in Burkina Faso represent about 30,000 t yr^–1^, costing about 22 billon FCFA (about US$40 million) for feed imports (Hamadou et al., 2005). To reduce Burkina Faso's dependence on feed imports, the government has encouraged the establishment of feed companies for the production of commercial feeds and cottonseed cake to increase the in‐country feed supply and to reduce the recurrent feed gap. However, locally produced commercial feeds account for only 23% of the country's feed need (FAO, 2019).

Cultivation of improved forages and collection and baling of native pasture species for making hay or haylage were promoted to livestock producers by governmental and nongovernmental organization agricultural extension services to mitigate the country's dry‐season feed gap. However, adoption of improved forages by livestock producers is very low due to inadequate access to seed; the lack of knowledge of forage production techniques; and the lack of resources, including finances, labor, time, and land (Gebremedhin et al., 2003; Kiema et al., 2012; Mapiye et al., 2006). The use of native pasture for grazing and straw collection for dry season feeding is currently limited due to decreasing natural pasture acreage and rangeland productivity. Furthermore, the increased use of these traditional practices by large numbers of livestock keepers in the country can cause biodiversity losses and soil fertility decline and degradation if not well managed. Therefore, these traditional feeding practices are not sustainable under the current context.

**Core Ideas**

- Livestock keepers in urban areas of Bobo‐Dioulasso are willing to pay for cultivated fodder.
- Willingness to pay for improved fodder is affected by the price of cottonseed cake.
- Producing quality fodder as a cash crop is an alternative for bridging the recurrent feed gap.
- Dissemination of improved forages to market‐oriented crop farmers is recommended.

In parallel, informal fodder and feed markets are developing in the urban and peri‐urban areas of large cities in sub‐Saharan Africa (Ayantunde et al., 2014; Konlan et al., 2018; Sanou et al., 2011) where dairy production and livestock fattening are done to supply the urban demand for ASF (Dossa et al., 2011). In those markets, fodder sold includes cereal stover {millet [*Panicum miliaceum* L.], maize [*Zea mays* L.], and sorghum [*Sorghum bicolor* (L.) Moench]}; legume haulms {cowpea [*Vigna unguiculata* (L.) Walp.] and peanut [*Arachis hypogaea* L.]}; road‐side fresh fodder from native pasture species, such as bluestem (*Andropogon gayanus* Kunth), hippo grass [*Echinochloa stagnina* (Retz.) P. Beauv.], Kyasuma grass (*Pennisetum pedicellatum* Trin.), and itchgrass [*Rottboellia exaltata* (L.) L. f.]; browse species, such as barwood (*Pterocarpus erinaceus* Poir.)*, Piliostigma* sp., and applering acacia [*Faidherbia albida* (Delile) A. Chev.]; and other agricultural byproducts (Ayantunde et al., 2014; Konlan et al., 2018; Hamadou et al, 2005). However, no improved cultivated forage grasses are sold in those markets in Burkina Faso (Hamadou et al., 2005; Sanou et al., 2011).

Assuming that main buyers of fodder in those markets are the landless livestock producers, the sedentary former pastoralists in the big cities, and the transhumant pastoralists who experienced recurrent feed shortages, this study aims to investigate their WTP for cultivated fodder (dual‐purpose cereals and legumes and improved forage) if produced by third parties, especially by market‐oriented crop farmers.

To our knowledge, little to no effort has been devoted toward promoting improved forage to supply the high demand for feed in Burkina Faso, yet there is a great need for such forages due to the growing forage markets, growing demand for feed (Sanou et al., 2011), and low adoption of improved forage production among livestock keepers (Hamadou et al., 2005).

In the economic literature, WTP assessment for agricultural technologies and innovation diffusion into the livestock sector has been successfully used by several authors for improved forage seed use in Ethiopia (Lemi, 2015), the use of insects as feed in Kenya (Chia, et al., 2020), and various other agricultural technologies (Muriithi et al., 2021). Assessing WTP among livestock keepers is essential for better understanding the needs of fodder users and buyers and to provide scientific evidence of the incentives for market‐oriented fodder production. Improved fodder production can be a source of incomes and job opportunities for youth, women, and other fodder chain actors involved in the marketing of the fodder and feed resources (Sanou et al., 2011). To this end, the objectives of this study were (a) to identify characteristics of cultivated fodder and dual‐purpose crops that fodder buyers are interested in, (b) to assess the WTP for cultivated fodder and factors that influence fodder buyers’ WTP for cultivated fodder, and (c) to estimate how much would they be willing to pay for quality cultivated fodder.

## 2 MATERIALS AND METHODS

### 2.1 Theoretical framework

The Theory of Consumers guided this study (Breidert, 2007; Dannenberg & Estola, 2018). In this study, we used the contingent valuation method (CVM) to measure the WTP for cultivated fodder as a cash crop by peri‐urban livestock producers in Bobo‐Dioulasso, Western Burkina Faso.

### 2.2 CVM description

The CVM is used to estimate the value that a person places on a good or product. The CVM is often used to estimate the monetary values of products or services that are not traded in markets or products or services that do not have prices yet in markets.

Willingness to pay is defined as the maximum price a buyer is willing to pay for a given quantity of a product or service (Gall‐Ely, 2009). The CVM uses questionnaires in which individuals record their decisions to pay for the products or services after being informed about the hypothetical situation of the basket of those products or services targeted (FAO, 2000). Gall‐Ely (2009) stated that the WTP assessment approach can be classified into four categories: (a) those based on the measurement of real or revealed data in a market (revealed preferences), (b) those based on survey (revealed preferences), (c) those based on survey or established data (stated preferences), and (d) those based on an offer to purchase, known as “incentive compatibility.” There are several models that are applied to WTP assessment. Survey methods are subject to nonresponse bias and representativeness of respondents but have the advantages of being easy to handle and are a direct measure that applies to any type of product. However, they have the disadvantage of having a strong strategic overestimation bias and that the market is hypothetical and informational. The calculation of WTP should take into account the types of values given by the groups of individuals. Also, overestimation bias should be corrected before calculating the average WTP. Similarly, null values (zeros) will have to be treated according to an appropriate method.

There are two main families of models to analyze WTP responses (Ami & Desaigues, 2000). The first family treats zero values and strictly positive values in the same way. In this situation, the explained variable is either WTP, its logarithm, or a Box–Cox transformation of WTP. For the last two cases, the underlying economic model is different. The logarithmic or Box–Cox transformation is only applicable if we consider that individuals tend to overestimate their WTP and that the error follows a log‐normal distribution (Ami & Desaigues, 2000). The second family of models treats zero and strictly positive WTP values differently. This is a two‐stage model of Heckman (1976) and the Tobit model proposed by Tobin (1958). The latter family seems to be applicable to the analysis of the WTP of livestock producers because zero values and strictly positive values should be treated differently. However, both models start from the multiple linear model.

### 2.3 Tobit model

The Tobit model is the censored regression model:

$$y^{*}=x^{\prime }\beta +e$$

The Tobit model is used when the dependent variable is censored from below at zero.

$$y=\left\{\begin{array}{c} y^{*}\mathrm{if}y^{*}>0\\ 0\mathrm{if}y^{*}\leq 0 \end{array}\right.$$

The dependent variable can be also expressed as *y* = max(*y* ^*^, 0). The upper limit, lower limit, or two limit values need to be specified to estimate the model. The Tobit model is a combination of two models (Probit + Truncated regression)

The Probit model is for the discrete decision of whether or not *y* is zero or positive:

$$\mathrm{Prob}\left(y>0\right)=\Phi \left(x^{\prime }\beta \right)$$

The Truncated model is for continuous decision (for the quantity of *y*|*y* > 0):

$$E\left(y|y>0\right)=x^{\prime }\beta +\sigma \lambda \left(\frac{x^{\prime }\beta }{\sigma }\right)$$

The coefficients in the Probit and in the truncated regression are restricted to be the same as in the Tobit model. The Tobit model assumes normality like the Probit model.

### 2.4 Marginal effects

The latent variable can be expressed by [*dE*(*y* ^*^)]/*dx* = β; the marginal effect for the censored sample (with zeros and positive amounts) can be estimated by [*dE*(*y* ^*^)]/*dx* = βΦ[(*x*′β)/σ]. The marginal effect on the truncated sample (only positive amounts) can be obtained from

$$\frac{dE(y|y>0)}{dx}=\left[1-\frac{x^{\prime }\beta }{\sigma }\lambda \left(\frac{x^{\prime }\beta }{\sigma }\right)-\lambda {\left(\frac{x^{\prime }\beta }{\sigma }\right)}^{2}\right]\beta$$

### 2.5 Empirical framework

The empirical method used in this study is presented as follows:

$${\mathrm{CAP}}_{i}^{*}=\beta_{0}+\beta_{1}X_{i1}+\cdots \beta_{k}X_{ik}+\varepsilon_{i}=X_{i}\beta \;+\;\varepsilon_{i}$$

The latent (unobserved) variable CAP*_i_* ^*^ measures the true value assigned by farmer *i* to the forage grown. Considering that, this latent variable is explainable by a number of socio‐economic variables and an error ε*_i_*,

$$\mathrm{If}\;\;{\mathrm{CAP}}_{i}^{*}\leq 0,\;\mathrm{then}\;\;{\mathrm{CAP}}_{i}^{*}=0$$

In this case the observation is censored in 0, and CAP*_i_* ^*^ is not observed. On the other, hand if CAP*_i_* ^*^≥ 0, then CAP*_i_* = CAP*_i_* ^*^ is observed. Therefore, we need to estimate the conditional expectation of CAP*_i_* = CAP*_i_* ^*^; knowing the explanatory variables *X_i_* = (*X_i_*_1_, …, *X_ik_*):

$$E\left(\mathrm{CA}P_{i}|x_{i}\right)=\emptyset \left(\frac{x_{i}\beta }{\sigma }\right)\left(x_{i}\beta \;+\;\sigma \lambda_{i}\right)$$

with

$$\lambda_{i}=\frac{\varphi \left(\frac{x_{i}\beta }{\sigma }\right)}{\phi \left(\frac{x_{i}\beta }{\sigma }\right)}$$

where φand ϕare the density and function, respectively, of a centered normal distribution reduce.

The parameters of this equation are estimated by the maximum likelihood method. The choice of variables to include in the Tobit model was based on intuition and several research studies in the field of forage adoption. The Tobit model includes only true valuations (strictly positive WTP and true zeros) and excludes false zeros, the objective being to analyze the determinants of WTP. By definition, true zeros are the amounts provided by farmers who do not currently want the forage crop as a feed solution for their livestock. False zeros mean that the farmer wants to have the forage grown but has problems assigning a monetary value. These false zeros are later replaced by the average WTP.

In the literature, several socio‐economic variables influence the decision of farmers on the feeding of their livestock. The dependent variable in this study is the amount the livestock producer is willing to pay per kilogram of forage grown. This is a quantitative variable. The explanatory variables are:

1. Agricultural byproduct (mainly cottonseed cake) price (FCFA kg^–1^) measures the price the farmer is willing to pay for 1 kg of by‐products. It is a continuous quantitative variable. It is expected to have a positive influence on the amount the farmer will pay for the crop fodder.
2. Expenses for crop residue purchase (FCFA) measures the amount of money used by the livestock producers to purchase crop residues to feed a herd in 2017. This variable is also expected to have a positive influence on the herder's decision because a herder who is accustomed to spending money on crop residues will be inclined to spend money on crop fodder. This is a continuous quantitative variable.
3. The practice of international transhumance is also a defined variable in the selection equation. A herder practicing international transhumance will tend to have a low value for cultivated fodder in relation to the fodder potentially available along their grazing routes or sites. The practice of transhumance is expected to have a negative effect on the purchase decision and on the price of cultivated fodder.
4. The practice of fattening is a binary variable (yes/no) that verifies whether the livestock producer practices this activity. Fattening consists of keeping animals in stalls on the farm to feed them on the spot. Fattening would therefore positively influence the decision to buy the cultivated fodder because livestock producers seek solutions to feed their animals well.
5. Experience in livestock farming (the number of years of experience) is a quantitative variable that measures the number of years the farmer has been making decisions about feeding a herd. Livestock producers with greater experience are expected to pay for the forage grown because of the difficulty finding dry‐season forage.
6. Livestock keeper ethnicity is also a binary variable (yes/no) that is worth 1 if the herder is of the Peulh ethnicity and 0 if not. Nomadic livestock production is culturally linked to the Peulh ethnic group in Burkina Faso. This variable is expected to have a positive influence on the decision to buy fodder because the Peulh rarely cultivate fodder and therefore do not have free crop residues for their livestock.
7. Knowledge of fodder and forage crops is a binary variable (yes/no) that seeks to determine whether the herder knew about fodder crops before the surveyors came. Indeed, the herder who knows about forage crops would be inclined to assign an appropriate value. Also, the value that one gives to a good depends on the level of knowledge that one has about this good (advantages/limitations).
8. Daily grazing practices is also a binary variable (yes/no) that checks whether the herder takes animals to pasture. This practice is thought to have a positive influence on the price of cultivated fodder because the herder knows the hardships and difficulties associated with obtaining fodder in the dry season in the zone and would be inclined to value this fodder access option.
9. The practice of cut and carry, hay making, and storage is a binary variable (yes/no) that seeks to verify whether the livestock keeper mows hay and stores it for the dry season. This variable is supposed to have a negative influence on the price of cultivated fodder. Indeed, farmers who practice this activity no longer feel the need to buy cultivated fodder because they have an alternative solution for feeding livestock in the dry season.
10. The number of livestock owned (mainly for ruminants) is a discrete quantitative variable. It measures the number of ruminants owned by the herder. Oxen, sheep, and goats are the ruminants considered in this study. We hypothesize that farmers with high numbers of ruminants will be willing to purchase the fodder grown to maintain the animals during the period of feed shortage. This variable is assumed to have a positive influence on the price of the forage grown.

The model can therefore be summarized as follows:

$$\begin{array}{ccc} & & \mathrm{CA}P_{i}=\beta_{0}+\beta_{1}\mathrm{PRIXTOUR}17_{1}+\beta_{2}\mathrm{DEPRESI}17_{2}\\ & & +\;\beta_{3}\mathrm{TRANSINTE}R_{3}+\beta_{4}\mathrm{OBOVIN}E_{4}+\beta_{5}\mathrm{NEX}P_{5}\\ & & +\;\;\beta_{6}\mathrm{ETHNI}E_{6}+\beta_{7}\mathrm{CONFOURRAG}E_{7}\\ & & +\;\;\beta_{8}\mathrm{PATUR}E_{8}+\beta_{9}\mathrm{FAUCHCONSER}V_{9}\\ & & +\;\beta_{10}\mathrm{NCHEPTE}L_{10}+\varepsilon_{i} \end{array}$$

where β_0_, β*_i_*, and ε*_i_* represent the constant, the estimated parameters, and the model error, respectively.

The type, levels of measurement, and expected signs of the different variables used in the model are presented in Table 1.

**TABLE 1:** Variables used in the willingness to pay modeling and estimation

**Variables**	**Variable description**	**Variable type**	**Measurement units**	**Sign**
Willingness to pay for 1 kg of cultivated fodder	WTP	Continuous	Montant (FCFA kg^–1^)	Dependent variable
Price of cotton seed cake in 2017 (FCFA)	PRICECSC17	Continuous	Montant (FCFA kg^–1^)	+
Expenses on crops residues in 2017 (FCFA)	EXPCRI17	Continuous	Montant (FCFA kg^–1^)	+
Transboundary transhumance	TRANSTRSH	Binary	1 = yes, 0 = no	+
Farmer ethnic group	ETHNICITY	Binary	1 = yes, 0 = no	+
Fattening practice	FATCATLE	Binary	1 = yes, 0 = no	+
Number of years in livestock farming	LIVFARMEXP	Binary	1 = yes, 0 = no	+
Knowledge of forage crop	FORAGEEXPS	Binary	1 = yes, 0 = no	+
Practicing animal grazing	ANIMGRAZING	Binary	1 = yes, 0 = no	+
Practicing grass cutting and conservation	PRACCUTCARRY SERV	binary variable	1 = yes, 0 = no	–
Number of cattle owned	CATTLEOWNED	discrete variable	number	+

### 2.6 Study area description

**FIGURE 1:**
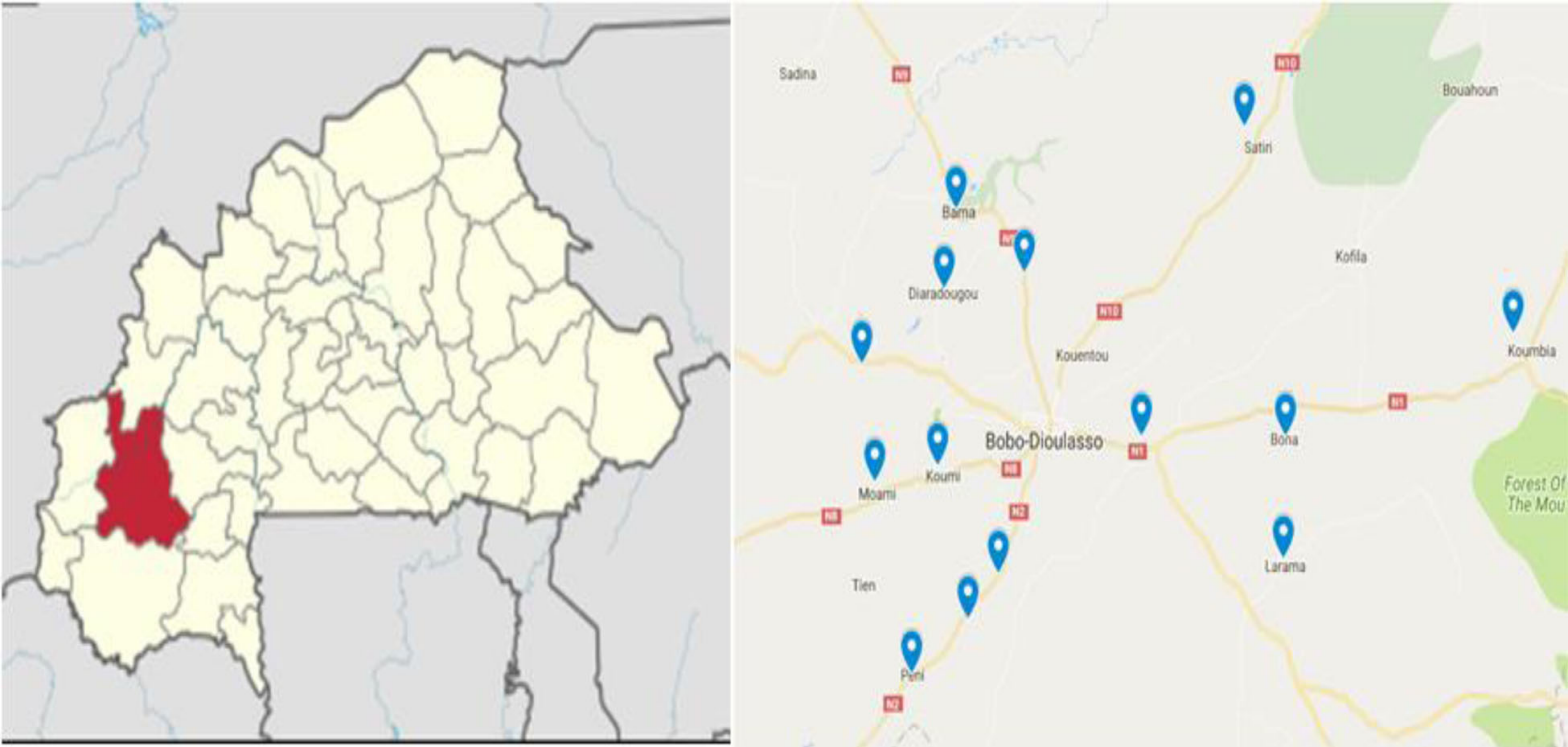
Study sites and respondents’ households’ location in the area

This study was conducted in the peri‐urban area of Bobo‐Dioulasso (Figure 1). The area is located in western Burkina Faso (11°11′ N, 4°17′ W; 405 m asl). Bobo Dioulasso is part of the south Sudanian agro‐ecological zone of Burkina Faso, which is part of Zone B of Köppen climate zone classification (Beck et al., 2018). The climate is characterized by mono‐modal distribution of wet season from May to October and dry season from November to April. The annual rainfall varies from 900 to 1,200 mm (Sawadogo, 2019). The mean annual rainfall of the last 10 yr was 1,049 ± 202 mm (Sawadogo, 2019). The daily mean temperature varies between 20 °C (minimum in December) and 35 °C (maximum in April), with mean annual temperature of 27 °C. Soils are mostly tropical feral soils (Bougma, 2013), and the natural vegetation is composed of open savanna and woodland, with several tree species as well as perennial and annual grasses (Zampaligré et al., 2021). Main agricultural production systems included integrated crop–livestock and agropastoral systems (Zampaligré & Fuchs, 2019) as well as urban and periurban livestock systems (dairy, beef, and poultry) (Dossa et al., 2011). Livestock species reared are mainly cattle, sheep, goats, and poultry (*Gallus domesticus*). Main food crops cultivated are sorghum [*Sorghum bicolor* (L.) Moench.], millet [*Pennisetum glaucum* (L.) R. Br.], rice (*Oryza sativa* L.), and maize (*Zea mays* L.). Cotton (*Gossypium hirsutum* L.), cowpea [*Vigna unguiculata* (L.) Walp.], and annual peanuts (*Arachis hypogaea* L.) are cultivated as cash crops. Fodder production among crop or livestock farmers is marginal despite efforts in promoting forage cultivation as livestock feed in the country (Cesar et al., 2005). Recent studies show increased use and sales of crop residues as feed, including roadside sales of collected fresh forages from natural pastures as well as commercial livestock feed such cottonseed cake, concentrates, and agricultural byproducts (Sanou et al., 2011).

### 2.7 Sampling

A stratified sampling method was used to estimate the farmers’ WTP for the forage. The list of farmers was obtained from the census database of the Ministry of livestock and Fisheries on animal agriculture households and enterprise in the study area.

For the purposes of the study, two strata were selected. The first stratum was urban and peri‐urban producers doing both livestock fattening and dairy production. These livestock farms are market oriented and located in the urban and peri‐urban areas, and their main products are meat (sale of live animals) and dairy. The second stratum is the proximity to the city of Bobo‐Dioulasso (Burkina Faso). The urban and peri‐urban livestock producers interviewed were located within a radius of 60 km of the city of Bobo Dioulasso. The sampling steps are as follows: (a) listing of each stratum and enumeration of the total population of the strata, (b) determination of the proportion of each stratum in relation to the total population, (c) investigating total size according to available resources, (d) determination of the sample of the strata to be investigated through their proportion in the total size of the strata, and (e) random selection of stratum members for data collection.

A total of 206 urban and peri‐urban livestock producers were selected for this study, and 202 of the selected producers were interviewed. This included semi‐intensive dairy farmers, livestock fatteners, and former pastoralists and agro‐pastoralists who settled in the vicinity of the city.

### 2.8 Data analysis

Descriptive statistics and econometric modeling were performed with STATA 14. The analysis consisted of estimating the Tobit model and its marginal effects (see section on theoretical, empirical frameworks, and Probit model description).

## 3 RESULTS AND DISCUSSION

### 3.1 Respondents’ socio‐demographic characteristics

A total of 202 respondents were interviewed for this study. The majority of interviewed households were male headed, with an average age of 45 yr. Their experience in livestock farming was 16 yr on average. The number of ruminants (cattle, sheep, and goat) owned ranged from 3 to 976, with an average of 87 animals per respondent. The majority of the livestock keepers are from Fulani/Peulh ethnic group (75%) and are actively involved in livestock husbandry in the vicinity of Bobo Dioulasso. Production systems are traditional pastoral and agro‐pastoral and peri‐urban semi‐intensive systems, with dairy and fattening being dominant (38.6 and 23.8%, respectively). Regarding feeding strategies, in addition to grazing on rangelands and crop residues mainly during the rainy season and post‐harvest season, respondents indicated that they also buy supplemental feeds (cottonseed cake, cereal and legume residues, agricultural by‐products [cereal brans], and other processed feeds during the dry season to feed their animals). Table 2 indicates the quantity and cost of purchased feed and fodder by respondents during 2017, which is the year before the interviews took place. Results showed that in 2017 each farm purchased an average of 1,300 kg of cottonseed cake, 290 kg of cereal straw (millet and sorghum), and 372 kg of legume haulms. Greatest expenses for feed were for cottonseed cake, followed by legume (cowpea and peanut) haulms and cereals straw, with estimated average costs per farm of 146,867, 20,589, and 14,793 FCFA, respectively (Table 2).

**TABLE 2:** Quantity and costs of feed and fodder purchased in 2017 by respondents for supplementing their animals during feed shortage periods

**Variables**	***n***	**Mean**	**SD**
Quantity of cottonseed cake purchased per farm in 2017, kg	200	1,328	10,631
Quantity of cereal straw purchased per farm in 2017, kg	192	290	862
Quantity of legume haulms purchased per farm in 2017, kg	197	372	1,034
Cost of cottonseed cake purchased per farm in 2017 (FCFA)	202	14,6867	185,907
Cost of cereal straw purchased per farm in 2017 (FCFA)	201	14,794	67,169
Cost of legume haulms purchased per farm in 2017 (FCFA)	193	20,589	30,573

*Note*. Source: survey data (2018).

### 3.2 Proportion of livestock producers willing to pay for cultivated fodder as cash crop

About 56% of respondents were willing to pay for cultivated forages to overcome their feed shortage and were able to propose a cost for 1 kg of cultivated forage. Another 23% of respondents were also willing to pay but failed to propose the amount they were able to pay. The majority of respondents (79%) were willing to pay for cultivated forage as cash crops in the study area.

Table 3 presents the price per 1 kg of cultivated quality forage that respondents were willing to pay for, along with key dependent variables.

**TABLE 3:** Descriptive statistics of the price per kilogram of fodder that the respondents were willing to pay for cultivated fodder relative to key dependent variables

**Variable**	**Modalities**	***n***	**Mean**	**SD**
Ethnic group of the respondents	Other groups	34	392	240
	Peulh/Fulani	79	246	375
Main livelihood activity	Crop and livestock	98	298	364
	Only livestock	15	237	194
Practice of fattening	No	81	329	393
	Yes	32	193	140
Dairy production	No	49	321	209
	Yes	64	267	421
Practice of herd grazing	No	6	640	1,095
	Yes	107	271	249
Practice of in country transhumance	No	72	337	397
	Yes	41	209	208
Practice of transboundary transhumance	No	103	301	354
	Yes	10	173	212
Number of animals	1–25 head	30	341	532
	≥25 head	83	272	248
Buyer of processed feed	No	11	137	186
	Yes	102	307	355

*Note*. Source: survey data (2018).

### 3.3 Amount that livestock producers were willing to pay for cultivated forages

The price (cost) for cultivated forage that respondents were willing to pay varied according to respondent and farm characteristics (Table 3). Surprisingly, results showed that integrated crop and livestock farmers were willing to pay more for cultivated forage than the livestock keepers. This might be due to the fact that crop and livestock farmers in the vicinity of big cities owned small land size, experienced feed shortage, and used purchased feed for supplementing their animals. In contrast, those whose main or only activity is livestock rearing are usually former pastoralists and agro‐pastoralists who use grazing strategies to feed their animals at low cost and therefore would not be willing to pay much for cultivated fodder. Respondents using mobility strategies (daily grazing and transhumance) to feed their animals are less likely to pay for cultivated fodder than those who are not practicing herd grazing and transhumance. In addition, as the size of the herd increased, respondents were willing to pay less for cultivated forage.

Livestock fatteners were willing to pay less than those who were not fattening their animals (Table 3). Purchase of feed and fodder, including concentrate, is common among livestock fatteners around big cities because most of them are landless market‐oriented livestock keepers who maximize profit by improving nutrition of the animals. Also, respondents who were already purchasing commercial feed to overcome their feed shortage were willing to pay three times more than those who were not using commercial feed.

### 3.4 Key factors influencing WTP for cultivated fodder by livestock producers

Table 4 presents results of the Tobit dual censorship estimates model. Because the price of cultivated fodder should not exceed the price of cotton cake for reasons of competitiveness, an upper limit was set. This limit is 200 FCFA kg^–1^ because the maximum price observed for the cottonseed cake over the last 5 yr was an average of 250 FCFA kg^–1^ in the dry season. The likelihood ratio at 10 degrees of freedom (likelihood ratio χ^2^ = 10) with a probability (Prob > χ^2^ = .000) indicates the model is significant at 1%. In this model, 89 observations are censored on the left, 62 observations are not censored, and 51 observations are censored on the right. Regarding factors influencing the cost farmers are willing to pay, the model shows that price of cottonseed cake and knowledge of cultivated fodder (forages and dual‐purpose crops) positively affects (*P* ≤ .001) the WTP for cultivated forages (Table 4). On the other hand, practicing transhumance and being a Fulani livestock producer (mainly former pastoralists and agro‐pastoralists but still doing extensive livestock practices) negatively affected the WTP (Table 4).

**TABLE 4:** Tobit model regression model results describing willingness to pay (WTP) as affected by various factors

	**Tobit dual censorship (ll = 0. ul = 200)**			**Marginal effects on WTP (censored at 0 and 200)**			**Marginal effects for truncated WTP (censored at 0 and 200)**		
**Variables**	**Coefficient**	***t***	***P* > *t***	***dy*/*dx***	***z***	***P* > *z***	***dy*/*dx***	***z***	***P* > *z***
Cotton seeds cake prices, 2017	0.056429***	5.62	0	0.024***	5.98	0	0.006***	4.93	0
Crop residue expenditures, 2017	−0.0001111	−0.46	.646	0	−0.46	.645	0	−0.46	.646
Transboundary transhumance	−111.79**	−2.49	.014	−47.717***	−2.58	.01	−12.313**	−2.45	.014
Ethnic group	−69.41114*	−1.89	.06	−29.628*	−1.9	.057	−7.645*	−1.85	.064
Practice of fattening	−28.10858	−0.81	.422	−11.998	−0.81	.417	−3.096	−0.81	.418
Number of years of experience	0.990793	0.73	.465	0.423	0.73	.463	0.109	0.73	.465
Knowledge of forage crops	137.89***	3.05	.003	58.858***	3.18	.001	15.188***	2.97	.003
Practice of grazing	−47.17054	−0.65	.516	−20.134	−0.65	.515	−5.196	−0.65	.515
Practice mowing and conservation	−9.985132	−0.28	.778	−4.262	−0.28	.778	−1.1	−0.28	.778
Livestock number	0.0024388	0.03	.978	0.001	0.03	.978	0	0.03	.978
Constant	6.601719	0.08	.94						
/sigma	169.4409								

*Note*. *n* = 202; likelihood ratio χ^2^ (10) = 114.77; Prob > χ^2^ = .0000; log likelihood = −486.88813; Nickname *R* ^2^ = .1054; 89 observations censored on the left at WTP ≤ 0; 62 uncensored observations; 51 observations censored on the right at WTP ≥ 200.

*Significant at the .05 probability level. **Significant at the .01 probability level. ***Significant at the .001 probability level.

To measure the effect of each variable on farmers' WTP for cultivated forage, an estimate of marginal effects was calculated. Table 4 shows the marginal effects under dual censorship; that is, for a WTP between 0 and 200 FCFA kg^–1^ and in a truncation situation (0 < WTP < 200). Key factors that influenced positively the WTP are the price of cottonseed cake in market (*P* ≤ .01), the knowledge of forage crops (*P* ≤ .01), and the ethnic group of respondents (*p* ≤ .10). The practice of transboundary transhumance negatively affected the WTP (*p* ≤ .001 for the dual censorship model and *p* ≤ .05 for the double truncation model).

The results of this estimation show that an increase of 100 FCFA of the price of cottonseed cake in the market will lead to an increase of 2.4 FCFA kg^–1^ for the cultivated fodder in the dual censorship model and 0.6 FCFA kg^–1^ for the double truncation model. In contrast, the practice of transboundary transhumance would result in a decrease in WTP of 47 and 12.31 FCFA kg^–1^, respectively, in the dual censorship model and in the double truncation model. For the knowledge of forage crops, it would generate an increase of 59 (dual censorship model) and 15 FCFA kg^–1^ (double truncation model). Being a Fulani livestock keeper would create a drop of 29 and 7 FCFA kg^–1^, respectively, for the dual censorship model and the double truncation model.

These results explicitly indicate that as the price of cottonseed cake supplement increases, farmers will be willing to pay more for cultivated fodder. This might be explained by the fact that cottonseed cake is the largest commercial feed supplement sold in Burkina Faso and the Sahel (FAO, 2014; ROEPA, 2012) In recent years, due to high demand for feed, there has been an increase of the price of cottonseed cake every year that varies depending on location in addition to the low availability of cottonseed produced in the country (FAO, 2014). The majority of buyers are the urban and peri‐urban market‐oriented dairy and livestock fattening farms. Productivity of those urban and peri‐urban livestock producers is constrained by limited access to quality fodder and by the low availability and high cost of commercial feeds such cottonseed cake; this may explain their willingness to pay for cultivated forage as an alternative to cottonseed cake.

In addition to cottonseed cake price, knowledge of cultivated forage was positively significant because ruminant producers know the role and importance of forage in ruminant nutrition, especially for dairy. Also, livestock farmers usually benefit from training in fodder production and ruminant feeding strategies by governmental and nongovernmental organization extension services to promote fodder cultivation, but landless producers lack an agricultural background, and most of them fail to produce their own forage even if it is needed. Exploring other actors (e.g., market‐oriented crop farmers, crop farmers cooperatives, youth, etc.) to produce fodder for market can be an alternative to supply quality fodder to the urban feed and fodder market. To our knowledge, except for crop residues (Ayantunde et al., 2014; Konlan et al., 2018), there are no cultivated forages (hay or silage) sold in the urban fodder markets in Burkina Faso and West Africa. However, this study shows that cultivating forage and baling as hay for market are likely to be adopted by market‐oriented livestock producers. Fresh collected pasture species hay and crop residues are already being sold in fodder markets in Bobo‐Dioulasso and Ouagadougou (Sanou et al., 2018, 2011).

The negative influence of daily grazing and transhumance practices and being a Fulani livestock keeper indicated that those farmers will continue to rely on exploitation of pasture and rangeland resources as feed for their animals. The reality is that the mobility strategy is being threatened by many constraints, such as expansion of agricultural fields, high taxes, obstruction of grazing areas and transhumance routes or corridors, and insecurity and social conflicts over access to water and forage resources (CORAF/WECARD, 2015). Today‘s transhumance practices require sufficient financial resources by the transhumants because there are many expenses to cover during transhumance, including tax payments, feed supplement purchases, and other costs for herders (food, communications, and health) and local traditional authorities (CORAF/WECARD, 2015; Thebaut, 2017). Also, there are many conflicts between crop farmers and livestock keepers, and the current insecurity and violent extremism in the Sahel are threatening the practice of livestock mobility, although governments have put in place some regulations for peaceful transhumance at local, national, and regional levels (CORAF/WECARD, 2015). Recent studies (Minot & Elahi, 2020; Thebaut, 2017) indicated that livestock keepers, including transhumant pastoralist and agro‐pastoralists, are increasingly purchasing supplemental feeds and fodder, including crop residues, in rural areas both within villages and territories and alongside the transhumance routes to overcome feed shortages during the long dry season. Thebaut (2017), in monitoring the cost of transhumance in the Sahel, found that the cost for feeds and fodder purchased during transhumance are among the top expenses of transhumant pastoralist along their movement in search for feed resources and water. A recent study conducted by Minot and Elahi (2020) among 1,000 rural households in Burkina Faso found that expenses for feed by livestock keepers in rural areas in Burkina Faso are the second largest investment in livestock production by rural crop and livestock farmers. These insights indicate that the extensive production systems, producers, including those practicing transhumant pastoralism who traditionally rely on mobility to feed their animals, are also investing in feed purchasing because fodder supply of communal pastures and rangelands is low, and access is constrained by the current escalating extreme violence in the Sahel. Therefore, alternatives for sufficient feed supply are needed to overcome feed shortages in the country and improve country livestock productivity to increase the supply of ASF to the cities and coastal countries where the demands are high. There is an opportunity for market‐oriented fodder producers to supply the growing demand for feeds and fodder both for peri‐urban market‐oriented livestock keepers and the extensive livestock systems in Burkina Faso and the Sahel. For this purpose, market‐oriented fodder production of dual‐purpose crops; perennial grasses, such *Brachiaria* cultivars; and appropriate valorization of the available crop residue biomass, such as rice, maize, millet, and sorghum straw, should be explored. Such cultivated forage and fodder production along with innovative feed technologies can be disseminated and upscaled to promote quality fodder production and therefore increase feed availability for Burkina Faso‘s livestock industry.

## 4 CONCLUSIONS

This study investigated the WTP for cultivated fodder as cash crops by peri‐urban livestock producers and the amount they are willing to pay. Results showed that the majority of respondents were willing to pay a relatively good price for cultivated fodder to feed their animals during feed shortage periods. High cottonseed cake prices and knowledge of cultivated fodder were positive factors driving this WTP; however, being engaged in transhumance negatively affected the WTP for cultivated fodder. This study showed that respondents were likely to be willing to pay for quality fodder if available in the market. This implies that policies that enable importing and growing such forages, raise awareness about their importance, and demonstrate best management practices for their production and marketing are needed in Burkina Faso. Market‐oriented fodder production and fodder business development, particularly by women and youth, should be promoted, and financial and other incentives as well capacity building should be provided to such entrepreneurs on using appropriate technologies and best management practices to optimize the growth, climate adaptation, nutritional quality, preservation, feeding, and marketing of cultivated forages. Such measures would help to increase the availability of quality feed, which is currently one of the critical issues of the livestock sector in Burkina Faso. In addition, these measures will help to meet the rising demand for ASF, particularly meat in Burkina Faso, and will generate jobs and incomes especially for the youth and women who critically need them.

## AUTHOR CONTRIBUTIONS

Ouédraogo Adama: Conceptualization; Data curation; Formal analysis; Methodology; Visualization; Writing‐original draft; Writing‐review & editing. Zampaligré Nouhoun: Conceptualization; Data curation; Formal analysis; Methodology; Visualization; Writing‐original draft, Writing‐review & editing. Balehegn Mulubrhan: Conceptualization; Funding acquisition; Methodology; Writing‐review & editing. Adesogan T. Adegbola: Conceptualization; Funding acquisition; Methodology; Project administration; Supervision; Writing‐review & editing.

## CONFLICT OF INTEREST

The authors declare no conflict of interest.
